# Infection of Keratinocytes with *Trichophytum rubrum* Induces Epidermal Growth Factor-Dependent RNase 7 and Human Beta-Defensin-3 Expression

**DOI:** 10.1371/journal.pone.0093941

**Published:** 2014-04-18

**Authors:** Yasemin Helene Firat, Maren Simanski, Franziska Rademacher, Lena Schröder, Jochen Brasch, Jürgen Harder

**Affiliations:** Department of Dermatology, University Medical Center Schleswig-Holstein, Campus Kiel, Kiel, Germany; Dr. Margarete Fischer-Bosch and University of Tübingen, Germany

## Abstract

Human keratinocytes are able to express various antimicrobial peptides (AMP) to protect the skin from exaggerated microbial colonization and infection. Recently, *in vitro* growth-inhibiting activity of the skin-derived AMP psoriasin, RNase 7 and human beta-defensin (hBD)-2 against dermatophytes such as *Trichophyton* (*T.*) *rubrum* have been reported. To evaluate whether keratinocytes are able to respond to *T. rubrum* infection by an induced expression of AMP we exposed primary keratinocytes to living conidia of *T. rubrum*. This led to conidia germination and mycelial growth which was paralleled by a strong gene induction of the skin-derived AMP RNase 7 and hBD-3. Gene expression of the AMP psoriasin (S100A7) and hBD-2 were only slightly induced. The *T. rubrum*-mediated RNase 7 gene induction was accompanied by increased secretion of RNase 7. Parallel treatment of the keratinocytes with *T. rubrum* and the cytokine combination IL-17A/IFN-γ resulted in synergistic induction of RNase 7 and hBD-3 expression. Since patients receiving therapy by inhibition of the epidermal growth factor receptor (EGFR) more often suffer from dermatophytoses we investigated whether EGFR may be involved in the *T. rubrum*-mediated RNase 7 and hBD-3 induction. Primary keratinocytes incubated with an EGFR blocking antibody as well as with the EGFR antagonist AG1478 showed a significantly diminished RNase 7 and hBD-3 induction upon exposure of the keratinocytes to *T. rubrum* indicating that EGFR is involved in the *T. rubrum-mediated* induction of RNase 7 and hBD-3. The growth of *T. rubrum in vitro* was inhibited by hBD-3 in a dose-dependent manner suggesting that hBD-3 may contribute to cutaneous innate defense against *T. rubrum*. Taken together our data indicate that keratinocytes are able to initiate a fast defense response towards *T. rubrum* by the increased expression of AMP active against *T. rubrum*. A dysregulation of AMP may contribute to chronic and recurring dermatophytoses.

## Introduction

Skin infections with dermatophytes belong to the most common infections worldwide [1], [2]. The resulting clinical manifestation of a skin infection with dermatophytes is called tinea. Typical causes of tinea are dermatophytes of the genera *Trichophyton*, *Microsporum* and *Epidermophyton* [1]. The pathogenesis of tinea is not completely understood. A characteristic of dermatophytes is their ability to adhere to the skin surface and to release a panel of enzymes which degrade host lipids and proteins. Especially the capability of dermatophytes to degrade keratin and to use it as a nutrient provides dermatophytes with an advantage to colonize the uppermost skin layers [3].

To establish an infection dermatophytes have to cope with the cutaneous innate defense system. Antimicrobial peptides (AMP) are important effector molecules of the innate skin defense due to their broad spectrum of potent antimicrobial activity allowing them to rapidly kill microorganisms [4]. Although the role of AMP to control the growth of bacteria on the skin surface and to protect the skin from infection is widely recognized, the role of AMP in tinea is less explored. Recently, it has been reported that AMP are induced in the lesional epidermis of tinea patients [5] and that the skin-derived AMP psoriasin, hBD-2 and RNase 7 are able to inhibit the growth of dermatophytes *in vitro* [6]. Thus, there is increasing evidence that AMP may also play an important role in the innate cutaneous defense against dermatophytes.

To gain further insight into the role of AMP in cutaneous innate defense against dermatophytes we analyzed whether keratinocytes challenged with the dermatophyte *Trichophyton* (*T.*) *rubrum*, the main cause of persisting dermatophytosis in humans [7], [8], respond through an induced expression of AMP. Our study revealed that primary keratinocytes exposed to *T. rubrum* showed a significant induction of the skin-derived AMP RNase 7 and hBD-3 mediated by the epidermal growth factor receptor (EGFR). In addition we report on the ability of hBD-3 to restrict the growth of *T. rubrum in vitro*. Together, these data strengthen the hypothesis that AMP may play a major role in cutaneous innate defense against infection with dermatophytes.

## Materials and Methods

### Culture and stimulation of primary keratinocytes

Foreskin-derived primary keratinocytes pooled from different individuals were purchased from PromoCell (Heidelberg, Germany) and cultured in Epilife medium (Life Technologies, Carlsbad, USA) at 37°C in a humidified atmosphere with 5% CO_2_. For stimulation experiments with conidia and cytokines, keratinocytes were seeded in 12-well tissue culture plates (3.8 cm^2^/well, BD Biosciences) and used at 100% confluence. Cytokines used for stimulation were from PeproTech (Rocky Hill, NJ). The EGFR-blocking antibody cetuximab was obtained from Merck (Darmstadt, Germany) and AG1478 was purchased from Enzo Life Sciences (Farmingdale, NY). Typical strains of *T. rubrum* isolates were obtained from the Department of Dermatology, University Hospital of Schleswig-Holstein in Kiel, Germany isolated from tinea lesions of patients who have given written consent which was approved by the Institutional Review Board of the University of Kiel. Patient data were anonymized and de-identified prior to analysis. The strains were identified by established morphologic and physiologic criteria [9]. Microconidia were prepared as recently described [6].

### RNA isolation and cDNA synthesis

After treatment, cells were washed with PBS and cells from one well of a 12-well plate were harvested and lysed using 500 µl Crystal RNAmagic reagent and total RNA was isolated according to the supplier's protocol (Biolab-Products, Bebensee, Germany). RNA quantity and quality was determined photometrically using a NanoDrop device (Peqlab, Erlangen, Germany) and 1 µg of total RNA was reversely transcribed to cDNA using oligo dT- primers and 50 Units Maxima Reverse Transcriptase (Thermo Fisher Scientific, Waltham, MA) according to the manufacturer's protocol.

### Real-time PCR

Real-time PCR analyses were performed in a fluorescence temperature cycler (StepOne Plus, Life Technologies) as previously described [10]. The following intron spanning primers were used: RNase 7 5′- GGA GTC ACA GCA CGA AGA CCA -3′ (forward primer) and 5′- CAT GGC TGA GTT GCA TGC TTG A -3′ (reverse primer); hBD-2 5′- GCC TCT TCC AGG TGT TTT TG -3′ (forward primer) and 5′- GAG ACC ACA GGT GCC AAT TT -3′ (reverse primer); hBD-3 5′- AGC CTA GCA GCT ATG AGG ATC -3′ (forward primer) and 5′- CTT CGG CAG CAT TTT CGG CCA -3′ (reverse primer); psoriasin 5′- AGA CGT GAT GAC AAG ATT GAG -3′ (forward primer) and 5′- TGT CCT TTT TCT CAA AGA CAT C -3′ (reverse primer). Standard curves were obtained for each primer set with serial dilutions of plasmids containing the amplification product. All quantifications were normalized to the housekeeping gene RPL38 (ribosomal protein L38) using the primer pair: 5′- TCA AGG ACT TCC TGC TCA CA -3′ (forward primer) and 5′- AAA GGT ATC TGC TGC ATC GAA -3′ (reverse primer). Relative expression is given as a ratio between target gene expression and RPL38 expression.

### ELISA

Secreted protein levels of RNase 7 were measured by ELISA as described [11]. The detection limit of the ELISA is 0.3 ng/ml. The secretion of hBD-3 using a hBD-3 ELISA was not detectable. This was most likely due to unspecific binding of the highly cationic hBD-3 to the surface of the 12-well plates because in control experiments we did not detect any hBD-3 (detection limit 0.4 ng/ml) using hBD-3 concentrations up to 100 ng/ml incubated for 24 h in the 12-well plates (without any cells).

### Growth inhibition assay

Culture of *T. rubrum*, isolation of conidia and analyses of growth inhibition of *T. rubrum* were performed as recently described [6] with minor variations. Germinating conidia were generated by incubation of conidia for 6 h at 37°C in Sabouraud's glucose broth (SAB B-D, BioMerieux, Lyon, France). Germinating conidia were then washed and resuspended in 10 mM sodium phosphate buffer (pH 7.2). 45 µl conidia suspension (111 conidia/µl) were incubated for 3 h at 34°C in a 96-well plate with 5 µl of 0.01% acetic acid containing different concentrations of hBD-3. After incubation, 150 µl Sabouraud's glucose broth containing 100 units/ml penicillin and 100 µg/ml streptomycin were added to each well and growth was monitored by measuring the optical density at 620 nm [6]. Each hBD-3 concentration was tested in triplicate. Recombinant hBD-3 was expressed in *E. coli* and purified as described [12] and stored in 0.01% acetic acid.

## Results

### Stimulation of primary keratinocytes with *T. rubrum* induces the expression of RNase 7 and hBD-3

**Figure 1:**
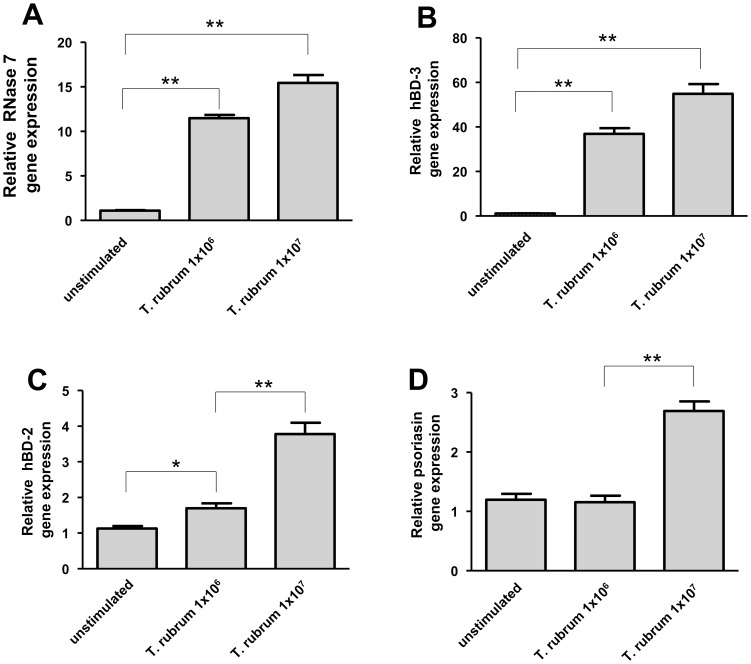
*Trichophyton rubrum* induces the expression of AMP in human keratinocytes. Primary keratinocytes were exposed to 1×10^6^/ml and 1×10^7^/ml conidia of *T. rubrum* for 24 h. Subsequently, gene expression of RNase 7 (a), hBD-3 (b), hBD-2 (c) and psoriasin (d) was analyzed by real-time PCR. Data are means ± SD (n = 3; *p<0.05, **p<0.01, Student's *t* test).

**Figure 2:**
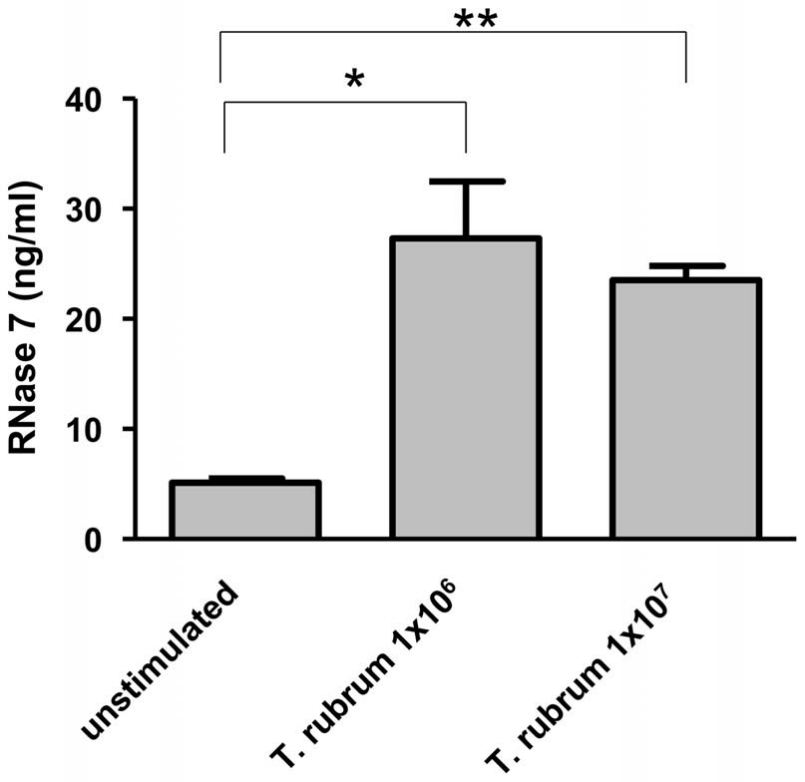
Infection of keratinocytes with *Trichophyton rubrum* leads to increased RNase 7 secretion. Primary keratinocytes were exposed to 1×10^6^/ml and 1×10^7^/ml conidia of *T. rubrum* for 24 h. Secretion of RNase 7 was determined by analysis of the presence of RNase 7 in the cell supernatants using ELISA. Data are means ± SD (n = 3; *p<0.05, **p<0.01, Student's *t* test).

To assess the capability of *T. rubrum* to induce the expression of AMP in keratinocytes we stimulated keratinocytes with 1×10^6^/ml and 1×10^7^/ml conidia of *T. rubrum* for 24 h. Incubation consistently triggered conidial germination and mycelia outgrowth as revealed by microscopic inspection. Stimulation resulted in a strong gene induction of RNase 7 and hBD-3 (Fig. 1a,b). In contrast, gene expression of hBD-2 and psoriasin was only weakly induced (Fig. 1c,d). Gene induction of RNase 7 correlated with increased RNase 7 protein secretion (Fig. 2).

### *T. rubrum* together with IL-17A/IFN-γ synergistically induces the expression of RNase 7 and hBD-3

**Figure 3:**
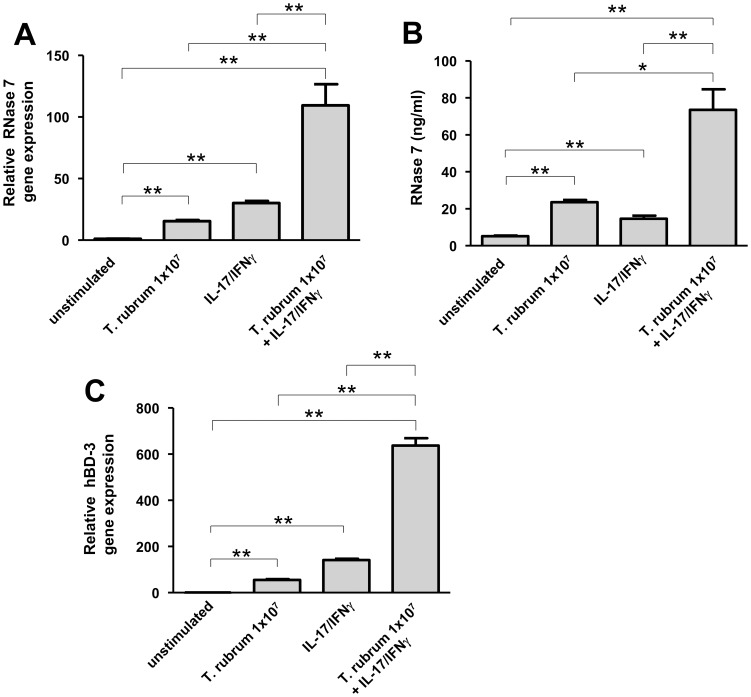
*Trichophyton rubrum* together with the cytokine combination IFN-γ/IL-17 synergistically induce RNase 7 and hBD-3 expression in keratinocytes. (A) Primary keratinocytes were incubated with 1×10^7^/ml conidia of *T. rubrum* together with IFN-γ/IL-17A (each 20 ng/ml) for 24 h. Gene expression of RNase 7 (a) and hBD-3 (c) was analyzed by real-time PCR. Release of RNase 7 was determined by ELISA (b). Data are means ± SD (n = 3; *p<0.05, **p<0.01, Student's *t* test).

It has been shown that the cytokine combination IL-17A/IFN-γ is a very potent inducer of AMP such as RNase 7 and hBD-3 expression in keratinocytes [13]. To analyze whether stimulation of keratinocytes with *T. rubrum* may influence the IL-17A/IFN-γ-induced RNase 7 and hBD-3 expression, keratinocytes were exposed to *T. rubrum* in the presence of IL-17A/IFN-γ. Gene expression of RNase 7 and hBD-3 was synergistically induced by *T. rubrum* and IL-17A/IFN-γ (Fig. 3a,c). Accordingly, secretion of RNase 7 was also synergistically induced (Fig. 3b).

### Epidermal growth factor receptor mediates the *T. rubrum*-induced expression of RNase 7

**Figure 4:**
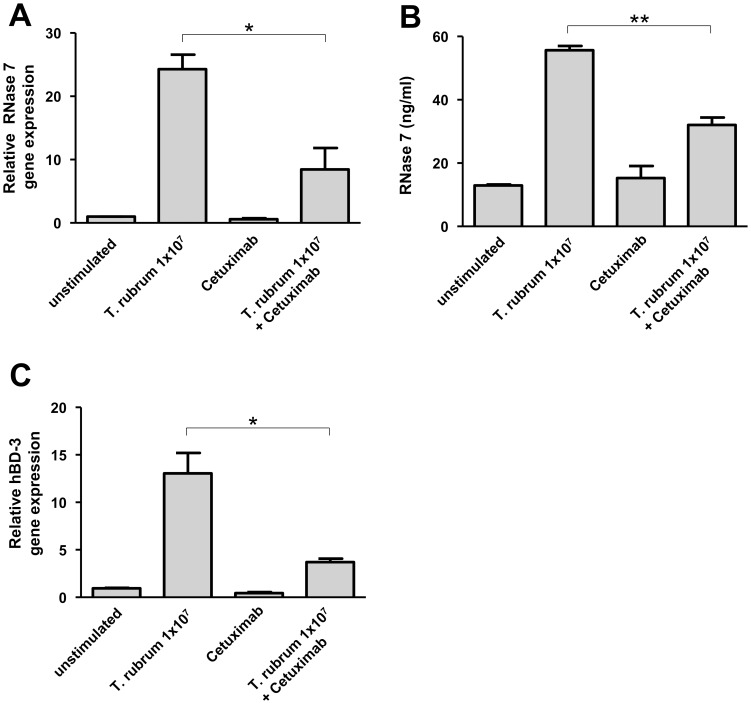
An antibody towards the epidermal growth factor receptor (EGFR) inhibited the *Trichophyton rubrum*-induced RNase 7 and hBD-3 expression in keratinocytes. Primary keratinocytes were pre-incubated with the EGFR-antibody cetuximab (20 µg/ml) followed by treatment for 24 h with 1×10^7^/ml conidia of *T. rubrum* in the presence of cetuximab (20 µg/ml). Gene expression of RNase 7 (a) and hBD-3 (c) was analyzed by real-time PCR. Release of RNase 7 was determined by ELISA (b). Data are means ± SD (n = 3; *p<0.05, **p<0.01, Student's *t* test).

It has been shown that therapeutic inhibition of the epidermal growth factor receptor (EGFR) is often associated with an increased occurrence of skin infections including infections with dermatophytes [14]. To investigate whether EGFR may play a role in the observed *T. rubrum*-induced RNase 7 and hBD-3 expression we used an EGFR-blocking antibody (cetuximab). Inhibition of the EGFR by this antibody led to a significant attenuation of RNase 7 and hBD-3 gene induction in primary keratinocytes upon stimulation with *T. rubrum* (Fig. 4a,c). Accordingly, analysis of RNase 7 secretion using ELISA revealed a significant decrease of *T. rubrum*-mediated RNase 7 secretion (Fig. 4b).

**Figure 5:**
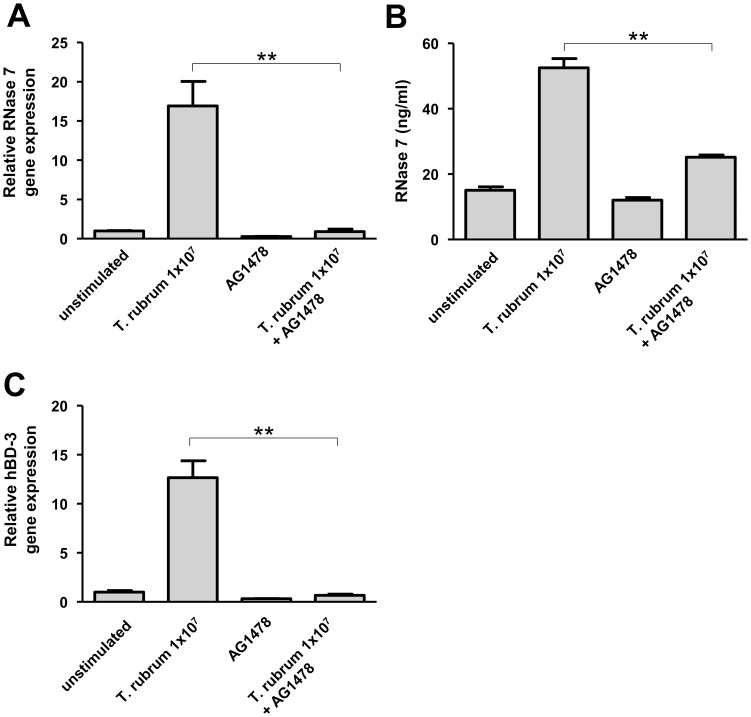
The EGFR antagonist AG1478 attenuates the induction of RNase 7 and hBD-3 in *Trichophyton rubrum*-infected keratinocytes. Primary keratinocytes were pre-treated with AG1478 (10 µM in 0.1% DMSO) followed by stimulation with 1×10^7^/ml conidia of *T. rubrum* with AG1478 (10 µM in 0.1% DMSO) for 24 h. Incubation with 0.1% DMSO served as a control. Gene expression of RNase 7 (a) and hBD-3 (c) was analyzed by real-time PCR. Release of RNase 7 was determined by ELISA (b). Data are means ± SD (n = 3; *p<0.05, **p<0.01, Student's *t* test).

To further evaluate the influence of EGFR we used the EGFR antagonist AG1478, a tyrosine kinase inhibitor selective to EGFR. In concordance with the cetuximab-experiments the use of AG1478 strongly inhibited the RNase 7 gene and protein induction in primary keratinocytes following treatment with *T. rubrum* (Fig. 5a,b). Similarly, *T. rubrum*-mediated hBD-3 gene induction was also inhibited by AG1478 (Fig. 5c)

### HBD-3 restricts the growth of *T. rubrum*

**Figure 6:**
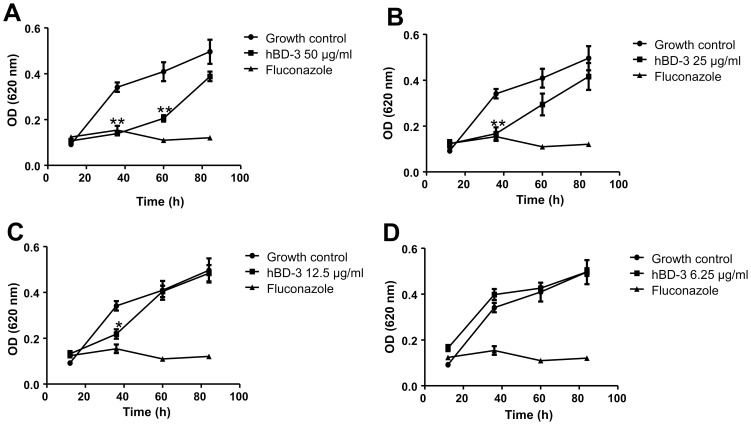
HBD-3 restricts the growth of *Trichophyton rubrum*. 45 µl of germinating conidia of *T. rubrum* were incubated in a 96-well plate for 3 h at 34°C in 10 mM sodium phosphate buffer (pH 7.2) together with 5 µl of hBD-3 (solved in 0.01% acetic acid) to reach an end concentration of 50 µg/ml hBD-3 (a), 25 µg/ml hBD-3 (b), 12.5 µg/ml hBD-3 (c) and 6.25 µg/ml hBD-3 (d). Incubation without hBD-3 (only 5 µl 0.01% acetic acid) served as a growth control and treatment with the antifungal drug fluconazole (100 µg/ml end concentration) served as a positive control. After 3 h incubation 150 µl Sabouraud's glucose broth containing 100 units/ml penicillin and 100 µg/ml streptomycin was added to each well and growth was monitored over time by measuring the optical density at 620 nm. Data are means ± SD of one representative experiment of two, each performed in triplicate samples (*p<0.05, **p<0.01 compared to growth control, Student's *t* test).

Since we observed a strong gene expression of hBD-3 in *T. rubrum*-stimulated keratinocytes and because a potential activity of hBD-3 against *T. rubrum* or other dermatophytes has not yet been elucidated we questioned whether hBD-3 may influence the growth of *T. rubrum*. Therefore germinated conidia of *T. rubrum* were incubated for 3 h with hBD-3 followed by incubation in Sabouraud's glucose broth. The growth of *T. rubrum* over time was assessed by densitometrical analysis as recently reported [6]. The results of this experiment revealed that hBD-3 dose-dependently inhibited the growth of *T. rubrum* (Fig. 6a–d). Concentrations of 50 µg/ml to 12.5 µg/ml hBD-3 significantly inhibited the growth of *T. rubrum* monitored after 36 h (Fig. 6 a–c). Thus, hBD-3 efficiently restricted the growth of *T. rubrum*.

## Discussion

Keratinocytes are the first cells dermatophytes have to cope with when they start to colonize and infect skin. A main defense reaction of keratinocytes against invading microorganisms is the induced expression of antimicrobial peptides (AMP) [4]. AMP represent an evolutionary well conserved host defense strategy to rapidly and efficiently control the growth of microbes due their broad spectrum antimicrobial activity [15], [16].

In this study we show that primary keratinocytes exposed to the most common dermatophyte *T. rubrum* [7], [8] strongly boost the expression of the AMP RNase 7 and human beta-defensin (hBD)-3. These data are in concordance with a recent study reporting an increase of immunoreactivity against the AMP hBD-2, -3, RNase 7 and psoriasin in the lesional epidermis of patients suffering from tinea [5]. The *T. rubrum*-induced RNase 7 and hBD-3 expression was synergistically increased in the presence of the cytokines IFN-γ and IL-17A, a cytokine combination recently shown to be highly efficient in inducing AMP in keratinocytes [13]. These data indicate that keratinocytes are able to sense dermatophytes leading to the induction of AMP. The identity of the responsible pattern recognition receptor(s) remains to be shown. Toll-like receptors (TLRs) such as TLR-2 may be possible candidates. TLR-2 has been implicated in the recognition of the yeast *Candida albicans* [17]–[19] and the expression of TLR-2 and TLR-4 is upregulated in tinea [5]. Other potential candidate receptors could be the C-type lectin-like receptors dectin-1 and dectin-2 which both have been implicated into the recognition of fungi [20], [21] including *Trichopyton rubrum* [22]. Dectin-1 mutations were described that reduce recognition of fungal β-glucan and thereby facilitate mycotic infections including onychomycosis caused by *T. rubrum* [23].

Recently, *in vitro* experiments demonstrated that the skin-derived AMP psoriasin, hBD-2 and RNase 7 were able to restrict the growth of *T. rubrum* [6]. In the present study we identified hBD-3 as an additional potent AMP to inhibit the growth of *T. rubrum*. The activities of hBD-3 and RNase 7 against *T. rubrum* and their induced expression in *T. rubrum*-treated keratinocytes as well as their upregulation in lesional skin of tinea patients suggest that AMP such as RNase 7 and hBD-3 may play a crucial role in the initial defense reaction of keratinocytes against invading dermatophytes. Deficiencies in the AMP-based cutaneous innate defense against dermatophytes may increase the susceptibility to develop tinea, a hypothesis which needs to be proven in further studies.

It has been reported that patients receiving therapy by inhibition of the EGFR often suffered from cutaneous infections including dermatophytoses [14]. Therefore we wondered whether an inhibition of the EGFR may negatively affect AMP induction in keratinocytes infected with dermatophytes. Indeed, blocking the EGFR resulted in a significantly decreased hBD-3 and RNase 7 expression in keratinocytes exposed to *T. rubrum*. These results make it likely that an attenuation of AMP expression through the anti-EGFR therapy may contribute to the observed increased dermatophytes infection rate in these patients, a hypothesis which needs to be proven in further studies. Future studies have to elucidate the molecular mechanisms and involved signal transduction pathways leading to activation of the EGFR by *T. rubrum*. Our results are in concordance with recent data reporting that keratinocytes treated with the EGFR-inhibitor erlotinib showed a decreased expression of AMP and a reduced antimicrobial activity of conditioned medium of primary human keratinocytes against *Staphylococcus aureus* [24].

In summary, our data indicate that an exposure of keratinocytes to *T. rubrum* leads to the induced expression of the AMP RNase 7 and hBD-3 in an EGFR-dependent manner. Increased level of AMP may help the host to control the growth and spread of *T. rubrum* and probably of other dermatophytes as well. A failure to adequately induce AMP to fight off dermatophytes may be responsible for the development of dermatophytosis as seen in patients with anti-EGFR treatment. Future studies have to evaluate whether a correlation between dysregulation of AMP expression and susceptibility to dermatophytes exists.
